# A haplotype-resolved chromosome-level assembly and annotation of European hazelnut (*C. avellana* cv. Jefferson) provides insight into mechanisms of eastern filbert blight resistance

**DOI:** 10.1093/g3journal/jkae021

**Published:** 2024-02-07

**Authors:** Samuel C Talbot, Kelly J Vining, Jacob W Snelling, Josh Clevenger, Shawn A Mehlenbacher

**Affiliations:** Department of Horticulture, Oregon State University, 4017 Agriculture and Life Sciences Building, Corvallis, OR 97331, USA; Department of Horticulture, Oregon State University, 4017 Agriculture and Life Sciences Building, Corvallis, OR 97331, USA; Department of Horticulture, Oregon State University, 4017 Agriculture and Life Sciences Building, Corvallis, OR 97331, USA; Hudson Alpha Institute for Biotechnology, 601 Genome Way Northwest, Huntsville, AL 35806, USA; Department of Horticulture, Oregon State University, 4017 Agriculture and Life Sciences Building, Corvallis, OR 97331, USA

**Keywords:** chromosome-level, haplotype-resolved, *Corylus*, European hazelnut, genome, fungal disease resistance genes

## Abstract

European hazelnut (*Corylus avellana* L.) is an important tree nut crop. Hazelnut production in North America is currently limited in scalability due to *Anisogramma anomala*, a fungal pathogen that causes Eastern Filbert Blight (EFB) disease in hazelnut. Successful deployment of EFB resistant cultivars has been limited to the state of Oregon, where the breeding program at Oregon State University (OSU) has released cultivars with a dominant allele at a single resistance locus identified by classical breeding, linkage mapping, and molecular markers. *C. avellana* cultivar “Jefferson” is resistant to the predominant EFB biotype in Oregon and has been selected by the OSU breeding program as a model for hazelnut genetic and genomic research. Here, we present a near complete, haplotype-resolved chromosome-level hazelnut genome assembly for *“*Jefferson”. This new assembly is a significant improvement over a previously published genome draft. Analysis of genomic regions linked to EFB resistance and self-incompatibility confirmed haplotype splitting and identified new gene candidates that are essential for downstream molecular marker development, thereby facilitating breeding efforts.

## Introduction

European hazelnut (*Corylus avellana* L.) is an important specialty tree nut crop that is grown in temperate climates for use in the in-shell and kernel markets, typically consumed raw or roasted, in confectionaries and baked goods. The estimated value of the global hazelnut industry is three billion US dollars with Turkey representing nearly 70% of global production (Food and Agriculture Organization of the United Nations 2022). Hazelnut (2*n* = 2*x* = 11) is a woody perennial that is monecious, dichogamous, wind-pollinated, and self-incompatible (Hill *et al.* 2021). While all hazelnut species produce edible nuts, the European hazelnut (*C. avellana* L.) is the most widely grown because of its desirable characteristics such as large high-quality nuts, thin shells, and preferred flavor profile. Traditional cultivars are clonally propagated and originated as selections from the wild in Europe and western Asia (Mehlenbacher and Molnar 2021).

Commercial hazelnut production in North America has been limited due to the high susceptibility of European hazelnut to *Anisogramma anomala*, a biotrophic ascomycete that is the causal agent of eastern filbert blight (EFB) disease. *Anisogramma anomala* has coevolved with its endemic host, the American hazelnut (*Corylus americana*), and in the wild the disease is widely tolerated (Capik and Molnar 2012; Revord *et al.* 2020). Symptoms of EFB are apparent ∼18 months following initial infection and include branch die-back, girdling of trunks, and eventual tree and orchard death. While management techniques such as pruning, scouting for cankers, and applying fungicides can slow the disease's spread, they do not eliminate it (Pscheidt and Ocamb 2022). Thus, breeding for genetic resistance is considered the most sustainable approach to managing EFB.

Oregon State University (OSU) has been a leader in developing improved EFB resistant cultivars for the Pacific Northwest (PNW), where Oregon represents 95% of US hazelnut production. The OSU hazelnut breeding program's primary contribution to EFB-resistant cultivar development can be traced to a 1975 discovery in southwest Washington of the obsolete pollinizer, “Gasaway”, which was completely free of EFB in a highly infected and dying orchard of “DuChilly” (Thompson *et al.* 1996). To date, multiple resistant pollinizers and cultivars derived from “Gasaway” have been released (Mehlenbacher and Molnar 2021), and underlie the expansion of acreage planted in Oregon from ∼11,000 ha in 2009 to greater than 25,000 ha in 2022 (USDA National Agricultural Statistics Service 2022). Outside of Oregon, however, cultivars with “Gasaway” resistance have been shown to be susceptible to genetically diverse *A. anomala* populations (Muehlbauer *et al.* 2019). Indeed, a genome assembly of the pathogen has shown that it has one of the largest Ascomycota genomes, suggesting a high capacity for pathogenic variation (Cai *et al.* 2013). The long-term durability of Oregon's commercial hazelnut orchards and the potential for expanding hazelnut production is limited by the pathogen's variability and narrow resistance offered by “Gasaway”.

The availability of genomic resources in *Corylus* has been increasing in recent years. The cultivar “Jefferson” was chosen for the first *Corylus* genome assembly because it contains “Gasaway” EFB resistance, and it was selected from the reference mapping population (Mehlenbacher *et al.* 2006). However, the Illumina-based first draft was highly fragmented due to hazelnut's highly heterozygous nature and the limitations imparted by short-read sequencing and assembly technologies (Rowley *et al.* 2018). With advances in long-read sequencing, pseudo-chromosome level genome assemblies for *Corylus* have been made available for *C. avellana* cultivars “Tombul” and “Tonda Gentile delle Langhe” (Lucas *et al.* 2021; Pavese *et al.* 2021) and representative accessions of two other *Corylus* species, *C. heterophylla* Fisch (Liu *et al.* 2021; Zhao *et al.* 2021) and *C. mandshurica* Maxim (Li *et al.* 2021). However, these genome assemblies are collapsed and there has been no haplotype-resolved “phased” assembly that represents both homologous chromosomes. Distinguishing chromosome haplotypes is essential for determining the parental allelic contributions to self-incompatibility, EFB resistance, and other traits.

EFB resistance derived from “Gasaway” has been characterized as a dominant allele at a single locus with 1:1 segregation (Mehlenbacher *et al.* 1991, 2006). This source of resistance has been mapped to linkage group (LG) 6 using random amplified polymorphic DNA (RAPD) and simple sequence repeat (SSR) markers in a segregating population from a cross between two clones, EFB-susceptible “OSU 252.146”×EFB-resistant “OSU 414.062” (Mehlenbacher *et al.* 2006). From this mapping population, the elite cultivar “Jefferson” was identified for release and was the source of the first *Corylus* draft genome (Mehlenbacher *et al.* 2011; Rowley *et al.* 2012). Fine mapping of the “Gasaway” region using bacterial artificial chromosomes (BACs) identified a span of approximately 135 kb and five candidate EFB resistance genes (Sathuvalli *et al.* 2017). Other sources of EFB resistance have been identified and mapped in over 30 *C. avellana* cultivars and accessions, and while the majority map to LG6 (Sathuvalli *et al.* 2012; Colburn *et al.* 2015; Komaei Koma 2020), other sources of qualitative and quantitative resistance have been mapped to LG2 (Sathuvalli, Mehlenbacher, *et al.* 2011; Şekerli *et al.* 2021), LG7 (Sathuvalli *et al.* 2011; Bhattarai *et al.* 2017; Şekerli *et al.* 2021), LG10 and LG11 (Lombardoni *et al.* 2022), and more recently LG4 and LG1 (Mehlenbacher personal communication). A complete summary of resistant cultivars and their related linkage groups can be found in Table 1 of Mehlenbacher *et al.* (2023). The accurate identification of candidate genetic parental contributions underlying qualitative and quantitative loci for EFB resistance will significantly aid in selection across a diverse collection of *Corylus* germplasm, thereby allowing for development of cultivars with multiple resistance loci.

The largest class of characterized plant disease resistance (R) genes encodes N-terminal nucleotide binding site (NBS) and C-terminal leucine-rich-repeat (LRR) functional domains (McHale *et al.* 2006). The LRR domain is highly variable within and among plant species and is typically associated with direct or indirect pathogen effector protein interactions (Prigozhin and Krasileva 2021). R-genes are often localized into clusters within chromosomes and can have significant variations in encoded amino acid sequence motifs, even within specific categories of R-genes (Kroj *et al.* 2016; Bailey *et al.* 2018; Wang and Chai 2020). Extensive research conducted over the past two decades has demonstrated the successful deployment of NBS-LRR R-genes in a wide range of crops (Kourelis and van der Hoorn 2018). Investigating the complex molecular mechanisms of R-genes both within and across different plant species is an expensive and resource-intensive task. Past work has identified candidate EFB resistance genes in “Jefferson”; however, the functional descriptions are more than a decade old, and recent improvements in genome assembly, annotation algorithms, and curated databases of plant genomes represent an opportunity to improve the description of candidate R-genes. To better direct future research in the “Gasaway” resistance region, it is crucial to update the annotation of *Corylus* R-gene candidates and evaluate them for protein domain similarities. This analysis will offer insights into the putative functionality of these genes and help determine if other sources of EFB resistance share similar molecular components.

Hazelnut orchard design and elite cultivar development also require an understanding of self-incompatibility (SI). Hazelnut exhibits sporophytic self-incompatibility (SSI), whereby compatibility between cultivars is determined by the genotypes of the plants. Incompatibility is determined by a single highly polymorphic locus, with a minimum of two genes, one each for male and female identity. The best-characterized example of SSI is in *Brassica*, which consists of two genes related to pollen-stigma recognition: a female serine/threonine receptor kinase and a cysteine-rich protein that serves as the pollen's credentials for compatibility interactions (Schopfer *et al.* 1999; Takasaki *et al.* 2000). Both genes colocalize in clusters in the genome containing similar sequences, and the encoded proteins colocalize in the plasma membrane. These genes are thought to have evolved from pre-existing signaling systems related to pathogen defense (Zhang *et al.* 2011). To identify SI alleles in hazelnut, the current method is a time-consuming process that requires a library of tester pollens and fluorescence microscopy to visualize pollen germination (Mehlenbacher 1997); a total of 33 SI alleles have been identified thus far with a nine-level dominance hierarchy (Mehlenbacher 2014). The locus responsible for SI has been mapped to LG 5 (Mehlenbacher *et al.* 2006). Fine mapping of this locus revealed a 193 kb region containing 18 predicted genes that differentiate between two SI alleles, S_1_ and S_3_ (Hill *et al.* 2021). Previous studies have shown that *Corylus* displays a unique SSI mechanism and is independent of the well-characterized SSI system in *Brassica* (Hou *et al.* 2022). Remapping the SI locus will increase the precision of molecular marker development for SI alleles, enabling further investigation into the genic contributions from parental plants. This will also help reveal the molecular mechanisms involved in *Corylus* SSI and identify candidate genes responsible for SI specificity.

Here, we present a chromosome-length haplotype-resolved genome assembly and annotation of “Jefferson”. The assembly was produced using Pacific Biosciences HiFi reads and chromosome-scaffolded using high throughput chromosome conformation capture (Hi–C) sequence data. The practical value of this genome assembly is demonstrated by the separation of the two parents into haplotypes at the previously mapped locus for self-incompatibility alleles. Additionally, haplotype separation identified new candidate genes derived from the parent that contributed “Gasaway” EFB resistance, providing insight into the molecular mechanisms of resistance.

## Materials and methods

### Plant material

The *C. avellana* cultivars “Jefferson” and its parents, female “OSU 252.146” and male “OSU 414.062” were used for genome sequencing and assembly. “OSU 252.146” is susceptible to EFB and carries the SI alleles S_3_ and S_8_, whereas “OSU 414.062” has “Gasaway” resistance and is homozygous for the SI-allele S_1_. Young leaf material was collected from field grown trees in Corvallis, OR, USA. Plants were dark-caged for 2–3 days prior to leaf collection, and collected leaves were frozen in liquid nitrogen for Illumina, PacBio, and Hi-C sequencing. For same-day flow cytometry analysis, young leaf tissue was collected in the early morning of May 2020, from a field-grown tree of “Jefferson” following leaf budbreak. Flow cytometry reference material was collected the same day from young tomato leaf tissue (*Solanum lycopersicum* L. “Stupicke”) from 2-week-old potted plants grown in the greenhouse.

### DNA extraction, library preparation, and sequencing

PacBio library prep and sequencing were done at the University of Oregon Genomics & Cell Characterization Core Facility (GC3F). High molecular weight genomic DNA (gDNA) was extracted from 1 g of flash-frozen leaves using Circulomics Nanobind Plant Nuclei Big DNA kit (Part No. NB-900-801-001, PacBio, USA) following the Nuclei Isolation TissueRuptor protocol (Part No. 9002755, Qiagen, Germany). Extracted gDNA were all sheared to 20 kb with MegaRuptor 2 (Cat no. B06010002, Diagenode, Belgium). Standard HiFi libraries were prepped with SMRTbell Express Template isPrep Kit 2.0 and SMRTbell Enzyme Clean Up Kit 2.0 at 1× reaction scale (5 µg sheared gDNA input per sample), including overnight ligation with nonbarcoded SMRT bell adapters. Final HiFi libraries were size selected to omit fragments <10–12 kb using Sage Science BluePippin (BLU0001, Sage Science, USA). Two 8M SMRT cells were sequenced for “Jefferson” and HiFi reads were generated from the postprocessing of SMRTbell subreads according to PacBio default parameters (CCS.how). DNA extraction and Illumina library prep for parents, “OSU 252.146” and “OSU 414.062” were done in-house using a modified CTAB method (Xin and Chen 2012) and the iTRU library prep protocol (Glenn *et al.* 2019) and sequencing done at GC3F, according to then current Illumina HiSeq 4,000 protocols to generate 150 bp paired-end (PE) reads (Hill *et al.* 2021). For Hi–C sequencing, tissue processing, chromatin isolation, and library preparation was performed by Dovetail Genomics (Santa Cruz, CA, USA), with library preparation done according to Lieberman-Aiden *et al.* (2009). Parental Illumina reads were demultiplexed using the Stacks v2.0 Beta 10 process_radtags module (Rochette *et al.* 2019). Demultiplexed reads were checked for quality using FASTQC (version 0.11.5; Andrews 2010) and cleaned by removing adapters, trimming, and quality filtering using the BBTools software suite (Bushnell 2016). Reads associated with low-quality regions of flow cells that contained bubbles were removed with the BBmap script filterbytile.sh. BBDuk was then implemented to trim or remove contaminating iTRU adapters, keep PE reads larger than 130 bp, and reads above Q20.

### Flow cytometry

Flow cytometry was done on “Jefferson” using a propidium iodide (PI) staining technique (Dolezel and Bartos 2005). Solutions of nuclei extraction buffer and staining buffer for PI were prepared using the Cystain PI kit according to manufacturer protocols (Sysmex, Lincolnshire, IL, USA). Tomato (*Solanum lycopersicum* L. “Stupicke”) was used as a reference standard. The 2C DNA content of tomato has been determined to be 1.96 pg, where 1 pg DNA = 0.978 × 10^9^ bp (Dolezel and Bartos 2005). Absolute genomic DNA was calculated by the following formula:


$$\begin{array}{r} \mathrm{Sample}\;2C\;\mathrm{DNA}\;\mathrm{content}=\left[\frac{\left(\mathrm{sample}\;G1\;\mathrm{peak}\;\mathrm{mean}\right)}{\left(\mathrm{standard}\;G1\;\mathrm{peak}\;\mathrm{mean}\right)}\right]\;\times \\ \;\mathrm{standard}\;\;2C\;\mathrm{DNA}\;\mathrm{content}\;(\mathrm{pg}\;\mathrm{DNA}). \end{array}$$

Briefly, sliced leaf squares of tomato and “Jefferson” of equal size (∼0.5 cm^2^) were placed in a petri dish together before the addition of 0.5 mL of nuclei extraction buffer. The *C. avellana* samples and tomato standard samples were co-chopped for 30 s using a razor blade prior to filtering through a 30 μm nylon-mesh CellTrics into a 3.5 mL tube. Then, 2 mL of PI staining solution was added to the remaining tissue within the filter. The mixture was incubated at room temperature for 30 minutes inside a Styrofoam cooler to protect against light. Two replicated runs were conducted on different days to account for instrument variation. Stained nuclei were analyzed using a QuantaCyte Quantum P flow cytometer and CyPad software version 1.1. A minimum of 15,000 nuclei counts occurred before the manual gating of G1 sample and standard peaks for each run.

### Genome sequence assembly

An initial genome size was estimated with a *k-mer* analysis of HiFi reads using Jellyfish (version 2.3.0, RRID: SCR_005491) and the web version of GenomeScope2 (version 2.0, RRID: SCR_017014) with settings: *k-mer* length of 31 and read length of 15,000 bp (Marçais and Kingsford 2011; Vurture *et al.* 2017). A haplotype-resolved contig assembly was generated using hifiasm trio-partition algorithm (version 0.16.1-r375, RRID: SCR_021069; Cheng *et al.* 2021). First, individual *k-mer* counts of parental Illumina reads of the parents “OSU 252.146” and “OSU 414.062” were acquired using Yak (version 1.1) as input evidence for hifiasm trio binning. The Arima Hi–C mapping pipeline was followed to generate mapped Hi–C reads (github.com/ArimaGenomics/mapping_pipeline). YaHs (version 1.1, RRID: SCR_022965) was run independently on both haplotype assemblies produced by hifiasm with their respective Hi–C aligned, read-name sorted bam file (Zhou *et al.* 2022). A Hi–C contact map was generated for each haplotype. Contigs were combined and gap-filled using Juicebox (version 1.11.08, RRID: SCR_021172; Durand *et al.* 2017). Finalized Hi–C contact maps were curated by Hudson Alpha (Huntsville, AL, USA), using an unpublished Hi–C scaffolding and alignment tool that oriented “Jefferson” chromosomes based on the “Tombul” genome pseudo-chromosomal scaffolds (Lucas *et al.* 2021). To verify haplotype assignment accuracy, parental reads were realigned to each haplotype assembly. Final assembly metrics were generated by QUAST (version 5.0.0, RRID: SCR_001228; Mikheenko *et al.* 2018). Assembly completeness was assessed with BUSCO (version 5.4.6, RRID: SCR_015008) in genome mode, using the Embryophyta odb10 dataset (Manni *et al.* 2021). The quality of assembled repetitive genomic regions was assessed using the long terminal repeat (LTR) assembly index (LAI) metric; the data analysis pipeline was composed of LTRharvest within GenomeTools (version 1.6.1, RRID: SCR_016120), LTR_FINDER (version 1.2, RRID: SCR_015247), and LTR_retriever (version 2.9.4, RRID: SCR_017623) using suggested default parameters to predict and combine likely full length candidate LTR-RTs (retrotransposons) (Ou *et al.* 2018). Calculation of the LAI index was based on the formula: LAI = (intact LTRs/total LTR length)×100.

### Structural gene annotation

Gene prediction and annotation was facilitated by Illumina transcriptome data from the following sources: (1) “Jefferson” bark and leaf tissue, *C. avellana* “Barcelona” catkins, whole seedling of “OSU 954.076” × “OSU 976.091” including root tissue (Rowley *et al.* 2012) NCBI accession: PRJNA168381; (2) “Jefferson” style tissue (Sathuvalli *et al.* 2016); and (3) leaf bud tissue from *C. avellana* “Tombul”, “Çakildak”, and “Palaz”, publicly available from the National Center for Biotechnology Information (SRA: PRJNA316492; Kavas *et al.* 2020). The resulting set of reads putatively representing *C. avellana* was ∼423 million PE 150 bp RNA-seq reads. Similarly, a protein set consisting of 61,590 annotated proteins was curated from a previous unpublished version 3 “Jefferson” genome assembly and *C. avellana* “Tombul” (Lucas *et al.* 2021). Gene annotation was performed for both “Jefferson” haplotype assemblies. To create a repeat library of transposable element families, a RepeatModeler (RRID: SCR_015027) families set was concatenated with the haplotype-resolved chromosome-level assemblies of “Jefferson” and six other OSU *C. avellana* accessions that were trio-assembled using the same methods as “Jefferson” but without chromosome scaffolding (Talbot *et al.* personal communication). Low complexity DNA sequences and repetitive regions were soft masked prior to gene annotation using the default parameters of RepeatMasker (version 4.1.0, RRID: SCR_012954).

Structural annotations of protein-coding genes were identified using the gene prediction software AUGUSTUS, GeneMark-ES/EP+, and GenomeThreader, integrated by BRAKER1 and BRAKER2 (RRID: SCR_018964) (Stanke, Keller, *et al.* 2006; Stanke, Schoffmann, *et al.* 2006; Stanke *et al.* 2008; Li *et al.* 2009; Barnett *et al.* 2011; Gremme 2013; Lomsadze *et al.* 2014; Buchfink *et al.* 2015; Hoff *et al.* 2016, 2019; Brůna *et al.* 2020, 2021). First, BRAKER1 used a unique .bam file generated from the splice-aware aligner Hisat2 (Kim *et al.* 2019), of the previously described RNA-seq set aligned to each haplotype assembly. Second, BRAKER2 was run using the AUGUSTUS *Arabidopsis thaliana* training set and gene structures were predicted via spliced alignments with AUGUSTUS ab-initio and GenomeThreader integration on each masked haplotype genome using the combined protein dataset previously described. Gene predictions of the respective BRAKER1 and BRAKER2 haplotype runs were assessed for quality, deduplicated, and combined using TSEBRA with default settings (Gabriel *et al.* 2021).

To further improve this original gene annotation set, BRAKER3 was used (Gabriel *et al.* 2023). A new masked genome was generated for both haplotype assemblies using Extensive De Novo TE Annotator (EDTA) (version 2.1.0, RRID: SCR_022063) (Ou *et al.* 2019) with parameters: –anno 1 –cds –sensitive including the respective coding sequences and gene locations generated by the BRAKER1/BRAKER2 pipeline. Finalized gene prediction sets were produced using BRAKER3 that included soft-masked genomes, a curated Viridiplantae ODB11 protein set consisting of ∼5.3 million proteins, and the previously described RNA-seq dataset. BRAKER3 outputs were used as input for TSEBRA, with the -k parameter, to enforce and recover potential missing genes and transcripts produced by the BRAKER1/BRAKER2 pipeline.

### Functional gene annotation

Both haplotype annotation sets from TSEBRA were subject to predictive functional analysis using the transcript set within OmicsBox (version 3.0); the OmicsBox pipeline included CloudBLAST using BLASTx, InterPro, GO Merge, GO Mapping, and GO Annotation plus validation (Altschul *et al.* 1990; Götz *et al.* 2008; Paysan-Lafosse *et al.* 2022). Completeness of the predicted annotation sets was assessed using BUSCO –protein mode, inputting translated amino-acid sequences derived from CDS of gene transcripts and the Embryophyta odb10 dataset. To assess long-range structural variation between haplotype assemblies, translocations, inversions, and copy number variation were identified using minimap2 (version 2.23-r1111, RRID: SCR_018550) (Li 2018), and SyRI (version 1.6.3, RRID: SCR_023008) and visualized by plotsr (Goel *et al.* 2019; Goel and Schneeberger 2022). Conservation of putative high confidence homologs between assemblies were compared using OrthoFinder (version 2.5.4, RRID: SCR_017118) (Emms and Kelly 2019).

### Identification of candidate genes for EFB resistance and self-incompatibility

To identify potential disease resistance gene homologs, the amino acid sequence of annotated protein-coding genes from each assembly were queried against the Plant Resistance Gene Database (version 3.0) using DRAGO2-api (Osuna-Cruz *et al*. 2018). DNA alignments of previously identified RAPD and SSR marker sequence fragments, BAC-end libraries, and annotated protein-coding genes from “Jefferson” were aligned to the new genome assemblies using minimap2 (Li 2018). Marker locations were secondarily assessed for off-target allele-size amplification and multimapping by in silico PCR using each marker's corresponding primer pair mapped against the Jefferson V4 haplotype 1 and 2 genomes, allowing for 1–2 mismatches per primer pair. A multiple sequence alignment of the translated candidate R-genes from each haplotype was generated with MUSCLE (version 5.1.0, RRID: SCR_011812) using default settings (Edgar 2021). A phylogenetic tree of these sequences was created using the neighbor joining tree (BLOSUM62) calculation in JalView (Waterhouse *et al.* 2009). MEME software (version 5.4.1, RRID: SCR_001783) was utilized to identify conserved subdomains among the putative R-gene candidate proteins using the settings: -mod anr -nmotifs 10 -protein (Bailey *et al.* 2009).

In a similar approach, genes involved in self-incompatibility were remapped to both haplotype assemblies using previously identified fine-mapped markers and gene sets (Hill *et al.* 2021). These markers and genes served as query evidence in BLASTn/BLASTp searches of both haplotype assemblies. A multiple sequence alignment of the identified proteins of interest in each haplotype was generated using MUSCLE and visualized using the neighbor joining tree (BLOSUM62) within JalView. The complete genome assembly and annotation pipeline are summarized (Supplementary Fig. 1).

## Results and discussion

### Genome assembly

A combined total of 3.6 million PacBio HiFi reads with an average length of 15,597 bp were generated from two 8 M SMRT cells, resulting in 56.8 Gb of sequence data (∼147× genome coverage) (Supplementary Table 1). For the two parents, “OSU252.146” and “OSU414.062”, 295 and 218 million PE 150 bp Illumina reads were generated, yielding 44 Gb (115× coverage) and 32 Gb (85× coverage), respectively (Supplementary Table 1). These reads were used to generate hifiasm trio binned haploid genome assemblies spanning 385,825,918 bp and 372,534,284 bp, containing 663 and 229 contigs for haplotype 1 and 2, with N50s of 23.4 Mb and 22.5 Mb, respectively (Table 1).

**Table 1.** Summary statistics for the assembled *C. avellana* “Jefferson” genomes.

Statistics	“Jeff V4 Hap1”	“Jeff V4 Hap2”	“Jeff V4 Hap1” Chr-resolved	“Jeff V4 Hap2” Chr-resolved
Total scaffold number	663	229	11	11
Total assembly length (Mb)	385.8	372.5	349.7	352
N_50_ (Mb)	23.4	22.5	32.5	32.4
Largest contig (Mb)	34.0	38.9	48.25	47.6
Number of contigs merged	NA	NA	22	21
Number of predicted protein-coding genes	NA	NA	33,506	34,379

**Fig. 1.**
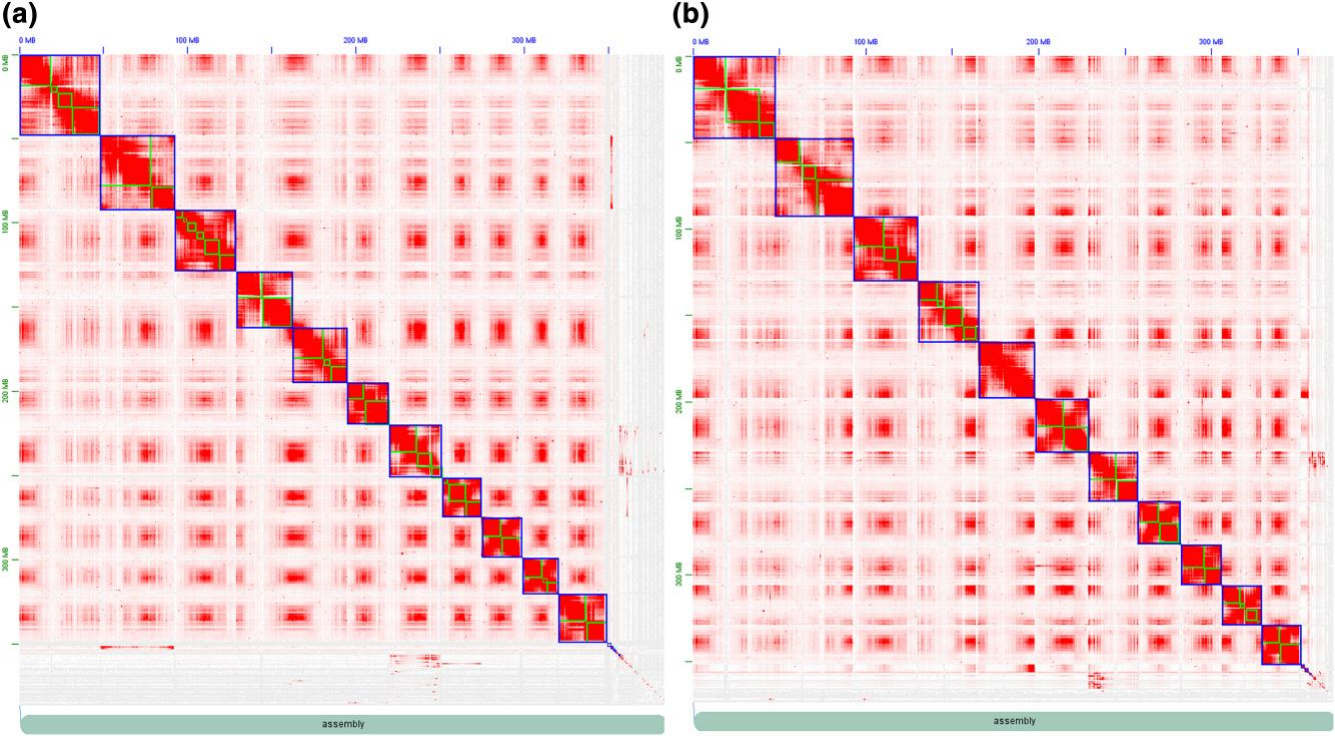
a) Hi–C interaction matrix for the “Jefferson” (*C. avellana*) haplotype 1 assembly. On the X and Y-axes is the distance in the genome assembly (Mb). The squares show contigs that are scaffolded within a larger square, representing a chromosome. Densely shaded areas within and outside the square indicate chromatin interaction loci, which are most abundant in chromosomes. In the lower right, unaligned contigs that lack sufficient Hi-C mapping depth are not incorporated into chromosomal scaffolds. b) Hi–C interaction matrix for “Jefferson” (*C. avellana*) haplotype 2 assembly.

The hifiasm haplotype assemblies were used as inputs to the chromosome scaffolding process. Hi-C sequencing of “Jefferson” generated ∼428 million PE 150 bp reads, for a total yield of ∼64.6 Gb (168 × coverage, Supplementary Table 1). The resulting “Jefferson V4' Hi-C scaffolded genome assemblies of each haplotype consisted of 11 pseudo-chromosomal scaffolds. The chromosome-level assemblies spanned a total length of 349,702,244 bp and 352,009,510 bp for haplotype 1 and haplotype 2, an N50 of 32.5 Mb and 32.4 Mb (Table 1, Supplementary Table 2). The Hi–C interaction matrix clearly differentiated between individual chromosomes in both haplotype assemblies wherein the diagonal pattern of the heat map shows that the frequency of interactions within a chromosome is high but low between chromosomes with no spill over (Fig. 1a and b). Alignment of parental reads to each genome assembly haplotype showed that the majority of reads from “OSU 252.146” aligned to haplotype 2, whereas the majority of reads from “OSU 414.062” aligned to haplotype 1 (Supplementary Table 3). BUSCO results in genome mode showed that both chromosome-level haplotype genome assemblies were of high, comparable quality and captured >97% of conserved genes in the Embryophyta dataset (Table 2).

**Table 2.** Assessment of genome completeness in “Jefferson” haplotypes using BUSCO.

Searching model	Protein categories	BUSCO			
		Haplotype 1		Haplotype 2	
		Number	Percentage	Number	Percentage
Genome	Complete BUSCOs (C)	1565	97.0	1575	97.5
	Complete and single-copy BUSCOs (S)	1535	95.1	1534	95.0
	Complete and duplicated BUSCOs (D)	30	1.9	41	2.5
	Fragmented BUSCOs (F)	6	0.4	6	0.4
	Missing BUSCOs (M)	43	2.6	33	2.1
	Total BUSCO groups searched	1614	100.0	1614	100.0
protein	Complete BUSCOs (C)	1572	97.4	1584	98.2
	Complete and single-copy BUSCOs (S)	1012	62.7	1008	62.5
	Complete and duplicated BUSCOs (D)	560	34.7	576	35.7
	Fragmented BUSCOs (F)	4	0.2	4	0.2
	Missing BUSCOs (M)	38	2.4	26	1.6
	Total BUSCO groups searched	1,614	100.0	1,614	100.0

### Genome size estimation

Flow cytometry was used to estimate a 1C genome size of “Jefferson” of 365.65 Mb (1C = 0.37 pg). This estimate is slightly smaller than a previously reported estimate of “Jefferson” (370 Mb; Rowley *et al.* 2018) and the reported range of other cultivars and diploid species in the subgenus *Corylus* (C*. cornuta*, *C. colurna*), which was between 1C = 0.41–0.43 pg (Bai *et al.* 2012; Vallès *et al.* 2014). PacBio HiFi reads of “Jefferson” were also input to GenomeScope2 to provide a secondary genome size estimate and heterozygosity of 348.9 Mb and 1.27%, respectively (Supplementary Fig. 2). The chromosome-resolved assemblies were four percent smaller than the flow cytometry prediction.

### Linkage map of “Jefferson”

The first available *C. avellana* linkage map was constructed using random amplified polymorphic DNA and simple sequence repeat (SSR) markers segregating in an F1 mapping population derived from a cross between “OSU 252.146” and “OSU 414.062”, the same population from which “Jefferson” was selected (Mehlenbacher *et al.* 2006). Since then, this linkage map has been improved by additional SSRs and data from a bacterial artificial chromosome (BAC) library (Sathuvalli *et al.* 2017; Mehlenbacher and Bhattarai 2018). To assign the linkage groups to pseudo-chromosomal scaffolds, 18 RAPD, 874 microsatellite, 4,100 paired BAC-ends with proper insert size, and 15,000 biallelic SNP marker sequence fragments were aligned to both Jefferson haplotypes using minimap2 (Li 2018), and compared to previous linkage mapping designations (Komaei Koma 2020). Both haplotypes were successfully assigned the same linkage group for each corresponding pseudo-chromosomal scaffold and renamed appropriately.

### Synteny of “Jefferson” haplotypes

**Fig. 2.**
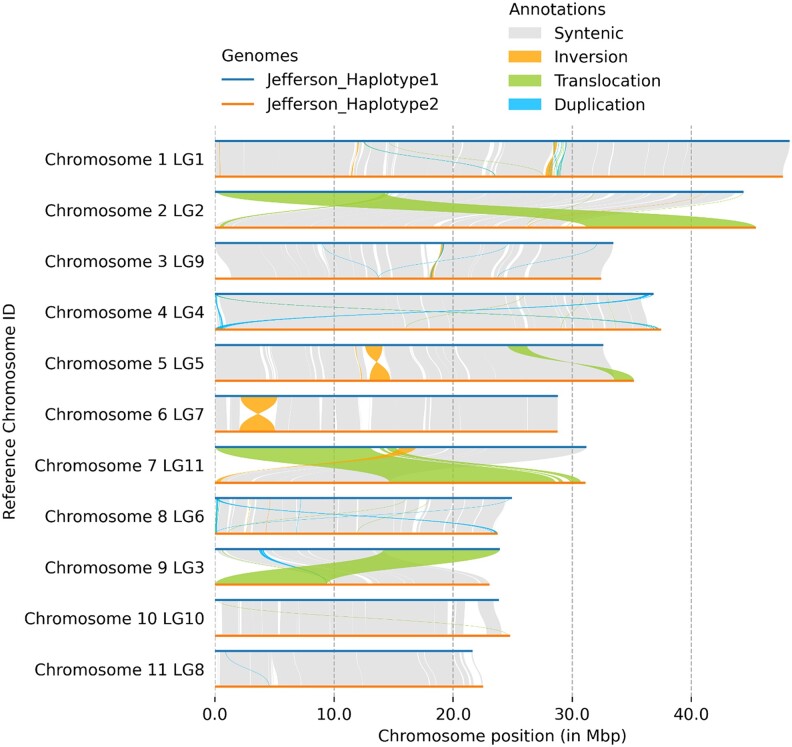
Synteny plot of the two “Jefferson” chromosome-resolved haplotype assemblies. Pseudo-chromosomal scaffolds of each haplotype were aligned to each other and labeled on the Y-axis with the chromosome ID and related linkage group. The X-axis shows the chromosome position and relative size in Mb. Chromosomes of haplotypes 1 and 2 are displayed as the top and bottom lines, respectively. Shading between the haplotypes represents synteny between genomic positions.

The “Jefferson” haplotype assemblies showed a high degree of synteny (Fig. 2). Differences in length between pseudo-chromosome haplotypes ranged from ∼16,000 bp (chromosome 6) to ∼2.5 Mb (chromosome 5); most scaffolds representing homologous chromosomes differed in length by an average of ∼892 kb. Between haplotypes there were three large scale translocations (chromosome 2, 7, and 9), two inversions (chromosome 5 and 6), and several small duplications, translocations, and gaps. The most notable of non syntenous regions were three large scale translocations on chromosomes two, seven, and nine, comprising total lengths of 14 Mb, 13 Mb, and 9.7 Mb, respectively. Despite nearly 93% of the haplotype assemblies mapping to one another, 33% of the alignments were categorized as having high divergence. Past cytological work has categorized three chromosome sizes, with two homologous pairs being large, five medium, and four small (Falistocco and Marconi 2013). Translocations have also been observed in *Corylus* (Salesses and Bonnet 1988). Reciprocal translocations are thought to frequently confound genetic map generation for many hazelnut populations (Lunde *et al.* 2006; Bhattarai *et al.* 2017; Marinoni *et al.* 2018) and are hypothesized to be the result of cytogenetic abnormalities, such as irregular chromosomal migration during cell division, or nondisjunction during microsporogenesis or megasporogenesis (Lagerstedt 1977). Mono-, bi-, and multi-valent chromosome pairings have been observed frequently in *Corylus* spp. and their hybrids (Woodworth 1929; Kasapligil 1963); this suggests that unequal crossover events may be common, especially when diverse germplasm is used. However, it is also possible these apparent translocations are errors from orienting the “Jefferson” Hi–C alignment against “Tombul”.

### Characterization of repeats

Prior to annotating protein-coding genes, genome repeat identification and masking was performed on the chromosome-level haplotype assemblies. The proportion of repeats and unknown elements identified in the initial RepeatModeler and RepeatMasker runs for the “Jefferson” haplotypes was higher than those reported for other *C. avellana* cultivars and *Corylus* species, with ∼65% of bases being masked. The high proportion of LTRs identified suggested potentially erroneous repeat calls that were introduced by the large concatenated LTR family's dataset. By rerunning the analysis using EDTA, a more stable view of LTRs was obtained, with 38.26 and 35.29% of repeats masked for haplotype 1 and 2 (Supplementary Tables 4 and 5). Class I retroelements made up 46–54% of all repeats identified for haplotype 1 and 2, respectively. *Gypsy* superfamilies were nearly double those of *Copia*, which is opposite of what has been previously reported in *C. avellana* “*Tombul*” but on par with *C. avellana* “Tonda Gentile delle Langhe” and silver birch (*Betula pendula*) “SB1” (Salojärvi *et al.* 2017; Lucas *et al.* 2021; Pavese *et al.* 2021). Nearly 20% of the total repeat length identified in either haplotype had LTRs categorized as “unknown”. The most significant difference observed between repeat elements of the haplotype assemblies was a doubling of the “repeat_region”, with 21 Mb and 9.5 Mb for haplotype 1 and 2, respectively. LAI analysis of haplotype 1 (LAI = 16.9) and haplotype 2 (LAI = 16.2), indicates that the repetitive and intergenic sequence space is of reference genome quality and a significant improvement from “Tombul” (LAI = 8.76) (Supplementary Fig. 3).

### Structural and functional gene annotation

In haplotypes 1 and 2, 32,431 and 33,159 protein-coding genes were identified and when considering alternative isoforms, these numbers increased to 48,832 and 50,663 coding transcripts, respectively. The protein-coding genes of both haplotype assemblies had an average length of 3,653/3,695 bp, with an average of 3.5 introns per longest isoform and median intron and exon lengths of 232 and 138 bp, respectively. For haplotypes 1 and 2, 21,201/21,354 (∼64%) of genes had no alternative isoforms, 7,767/8,089 (∼24%) had one alternative isoform and 3,453/3,716 (∼11%) had two or more isoforms. For each haplotype's predicted gene set, >97% of *C. avellana* genes were complete BUSCOs for the odb10 Embryophyta gene families (Table 2). Approximately 35% of highly conserved BUSCO genes were predicted as complete-duplicated, potentially due to alternative transcripts.

For haplotype 1, functional annotation analyses assigned GO terms and InterPro domains to 24,369 (72.7%) of transcripts. For the remaining transcripts in haplotype 1, 3,907 (11.4%) had no blast hits, 3,605 (10.8%) had only blast hits, 1,666 (5%) were identified with GO mapping. Similarly for haplotype 2, 24,932 (72.5%) of transcripts were assigned GO terms and InterPro domains. Of the remaining transcripts in haplotype 2, 3,907 (11.4%) had no blast hits, 3,725 (10.8%) had only blast hits, and 1,815 (5.3%) of transcripts were GO mapped (Supplementary Figs. 4 and 5). OrthoFinder was used to further characterize and assess conservation between predicted gene sets of each haplotype assembly. Of the combined 99,495 transcripts from haplotype 1 and 2, 96,193 (96.7%) were placed in a total of 31,779 orthogroups, with only 4,618 (4.6%) of genes being categorized as unique to a haplotype. To assess the overall distribution of disease resistance genes, DRAGO2 identified 3,620 and 3,659 putative genes with resistance-like domains for haplotype 1 and haplotype 2 assemblies. Many of these genes identified by DRAGO2 were receptor-like kinases and proteins (∼25%), with a small fraction being identified as NBS-LRRs (∼10%) (Supplementary Table 6).

### Potential candidate genes for self-incompatibility

The locus for pollen-stigma incompatibility was fine-mapped by Hill *et al.* (2021), who identified 18 genes within a 193.5 kb region on linkage group 5 that were associated with SI alleles S_1_ and S_3_. To remap the SI locus, BLASTn was used to align genes from the previous assembly to both chromosome-resolved haplotype assemblies of “Jefferson”. BLASTn searches returned twelve genes with 100% identity to the S_1_ allele among the newly predicted genes in haplotype 1, chromosome 5. In chromosome 5 of haplotype 2, 11 genes with 100% identity to the S_3_ allele were identified. Multiple genes that were previously identified as candidates for SI interactions in *Corylus*, PIX7 (*Putative interactor of XopAC_7_*) and MIK2 (*MDIS_1_-interacting receptor like kinase*) were also found in both “Jefferson” haplotypes. Haplotype 1 contained two copies of PIX7 and eight copies of MIK2, whereas haplotype 2 contained three copies of PIX7 and five copies of MIK2. The SI-locus occupied 86.6 kb in haplotype 1 and 222 kb in haplotype 2. The phasing of alleles within the chromosome 5 SI locus agrees with the previous fine mapping results showing that “OSU 252.146” contributed S_3_ to “Jefferson”, and is represented in the haplotype 2 assembly, whereas “OSU 414.062” which contributed S_1_ to “Jefferson”, is represented in the haplotype 1 assembly.

The similarity of PIX7 and MIK2 candidates was assessed using OrthoFinder, which assigned these genes to seven orthogroups. All seven PIX7 homologs were assigned to three orthogroups, whereas the majority of MIK2 homologs were assigned to a single orthogroup. This suggests that putative PIX7 and MIK2 candidate gene copies are highly conserved, but there may be some variation in protein subdomains that lead to the identification of multiple orthogroups. Indeed, of the eighteen genes identified as PIX7 or MDIS-1 homologs, all were variable in length (Table 3). Recent studies have shown that in *Brassica*, the most well-characterized SSI system, a small RNA is crucial for inducing methylation of recessive SI allele to induce compatibility (Yasuda *et al.* 2021).

**Table 3.** *Corylus avellana* “Jefferson” self-incompatibility homologs identified in the self-incompatibility region of both haplotypes of chromosome 5 (LG 5).

*Corylus avellana* gene	Amino acid length (bp)	Function
Hap1_g18435	513	Probable serine/threonine protein kinase PIX7
Hap1_g18437	695	MDIS1 interacting receptor like kinase 2 like
Hap1_g18438	328	MDIS1 interacting receptor like kinase 2 like
Hap1_g18439	937	MDIS1 interacting receptor like kinase 2 like
Hap1_g18441	357	MDIS1 interacting receptor like kinase 2 like
Hap1_g18442	767	MDIS1 interacting receptor like kinase 2 like isoform
Hap1_g18443	1,056	MDIS1 interacting receptor like kinase 2 like isoform
Hap1_g18444	112	Probable serine/threonine protein kinase PIX7
Hap1_g18445	177	MDIS1 interacting receptor like kinase 2 like isoform
Hap1_g18450	787	MDIS1 interacting receptor like kinase 2 like isoform
Hap2_g19113	477	Probable serine/threonine protein kinase PIX7
Hap2_g19115	417	MDIS1 interacting receptor like kinase 2-like
Hap2_g19117	937	MDIS1 interacting receptor like kinase 2-like
Hap2_g19118	182	Probable serine/threonine protein kinase PIX7
Hap2_g19119	950	MDIS1 interacting receptor like kinase 2-like
Hap2_g19124	793	MDIS1 interacting receptor like kinase 2-like
Hap2_g19138	1,296	MDIS1-interacting receptor like kinase 2-like
Hap2_g19148	513	Probable serine/threonine protein kinase PIX7

When considering the substantial number of SI alleles in *Corylus* (33 to date), it is possible that unannotated sRNA(s) are acting upon different variants of PIX7 or MIK2 to establish allelic dominance. Additional genomes of other *Corylus* cultivars with confirmed SI alleles will be needed to verify differences in SI alleles and putative candidate genes to further elucidate the complex molecular mechanism driving SSI and allelic hierarchy in *Corylus*.

### Potential candidate genes for EFB resistance in hazelnut

In “Jefferson”, EFB resistance is derived from “Gasaway” and is conferred by a dominant allele at a single locus that has been mapped between RAPD markers 152–800 and 268–580 on linkage group 6 (Mehlenbacher *et al.* 2006). Recent QTL (Quantitative Trail Loci) mapping in *C. americana* × *C. avellana* mapping populations associated LG6 EFB resistance in *C. avellana “*Tonda di Giffoni”, with SNP 93212 (Lombardoni *et al.* 2022). Aligning the associated paired-end sequences from SNP 93212 to “Jefferson” V4 haplotype 1 placed the QTL peak 20 kb upstream from the markers most closely associated with EFB resistance, and within BAC contig 43F13 in the fine-mapped region defined by Sathuvalli *et al.* (2017). When mapping the Sanger sequence of CC875206.1 W07-365 (365 bp), the RAPD marker originally extracted from the PCR band associated with W07 “Gasaway” resistance, the sequence is repeated 3 times in this region in both haplotypes of “Jefferson”; however, the sequence is truncated by ∼60 bp in haplotype 2 and spans an additional 100 kb in chromosomal space. Mapping the original Illumina reads from BAC 43F13 to both haplotypes revealed haplotype 1 as the source of the BAC contig and clearly defined the region coinciding with the associated BAC-end markers. The higher percentage of Illumina reads aligning to haplotype 1 from EFB-resistant parent “OSU 414.062” provides additional support for an EFB-resistance model with R-gene contributions derived from “Gasaway” present in haplotype 1 only.

Functional annotation of the “Jefferson” EFB resistance region on haplotypes of chromosome 8 (LG 6) identified several probable receptor-like kinases and putative disease resistance genes.

**Fig. 3.**
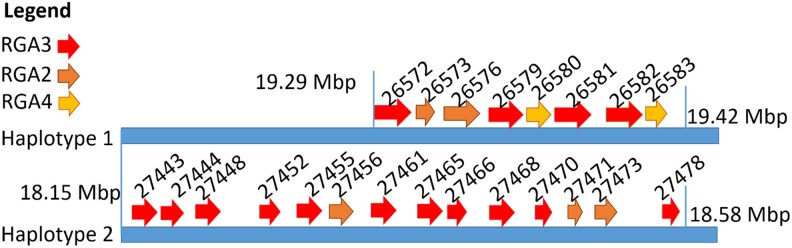
Putative EFB R-gene candidates (RGA-homologs) plotted on chromosome 8 of both haplotypes. Red arrows represent RGA3 homologs, orange arrows represent RGA2 isoform-X2 homologs and yellow arrows represent RGA4 homologs. The numeric gene ID for each respective homolog is listed above the arrow where haplotype 1 represents Hap1_g and haplotype 2 represents Hap2_g. Denoted as vertical lines in Mb are the start and stop positions of the R-gene cluster.

On haplotype 1, a region of approximately 125 kb contained five CNLs identified by DRAGO2 but eight genes with functional descriptions relating to “RGA” (Resistance Gene Analog). On haplotype 1, Hap1_g26572 and Hap1_g26573 were identified as having homology to RGA3 and a short 232aa RGA2-like isoform, respectively. Six other putative resistance genes were identified in haplotype 1, including a long 1,116 aa copy of disease resistance RGA2-like isoform in Hap1_g26576, three copies of RGA3 in Hap1_g26579, Hap1_g26581, and Hap1_g26582, and two copies of RGA4 in Hap1_g26580 and Hap1_g26583. Similarly, haplotype 2 contained fourteen genes with functional descriptions related to “RGA3” and “RGA2-like isoform” (Table 4), but only eleven were identified as CNLs by DRAGO2. None of the R-genes from haplotype 1 had a 100% match to haplotype 2 R-genes. In Fig. 3, the genomic location and orientation of the putative EFB R-gene candidates on chromosome 8 (LG6) are depicted for both haplotypes, showing that RGA3 homologs are closely linked to an RGA2-like isoform and an RGA4 homolog on haplotype 1, whereas R-gene candidates on haplotype 2 are identified as only RGA3 and one as RGA2-like isoforms, all ranging in distance from one another by 20–60 kb.

**Table 4.** *Corylus avellana* “Jefferson” candidate EFB r-gene homologs identified in the “Gasaway” resistance region locus on chromosome 8 (linkage group 6) of both haplotypes.

*Corylus avellana* gene	Amino acid length (bp)	Function
Hap1_g26572	1,213	Putative disease resistance protein RGA3
Hap1_g 26573	237	Disease resistance protein RGA2-like isoform X2
Hap1_g 26574	224	Cysteine-rich repeat secretory protein 38
Hap1_g 26576	1,116	Disease resistance protein RGA2-like isoform X2
Hap1_g 26579	891	Putative disease resistance protein RGA3
Hap1_g 26580	323	Putative disease resistance protein RGA4
Hap1_g 26581	1,191	Putative disease resistance protein RGA3
Hap1_g 26582	849	Putative disease resistance protein RGA3
Hap1_g 26583	274	Putative disease resistance protein RGA4
Hap1_g 26585	230	Cysteine-rich repeat secretory protein 38-like
Hap2_g27443	1,215	Putative disease resistance protein RGA3
Hap2_g27444	1,164	Putative disease resistance protein RGA3
Hap2_g27448	1,145	Putative disease resistance protein RGA3
Hap2_g27452	1,159	Putative disease resistance protein RGA3
Hap2_g27455	1,159	Putative disease resistance protein RGA3
Hap2_g27456	1,150	Disease resistance protein RGA2-like isoform X2
Hap2_g27461	1,178	Putative disease resistance protein RGA3
Hap2_g27465	1,145	Putative disease resistance protein RGA3
Hap2_g27466	847	Putative disease resistance protein RGA3
Hap2_g27468	1,159	Putative disease resistance protein RGA3
Hap2_g27470	472	Putative disease resistance protein RGA3
Hap2_g27471	725	Disease resistance protein RGA2-like isoform X2
Hap2_g27473	1,150	Disease resistance protein RGA2-like isoform X2
Hap2_g27478	473	Putative disease resistance protein RGA3

RGA4 has been characterized as an auto-inducer of immune response to the fungal disease rice blast, caused by *Magnaporthe oryzae* (*M. oryzae*), whereby RGA4 is tightly linked with RGA5, with the encoded proteins interacting as a homo and hetero dimer, such that both are required for resistance (Césari *et al.* 2014). Research suggests that the presence of an integrated heavy metal associated (HMA) domain within RGA5 mimics the pathogen effector target as a “decoy”, and upon direct binding to the effector, a signal is transduced to RGA4, relieving RGA4 repression and initiating an immune response (Xi *et al.* 2022). Heavy metal-associated isoprenylated plant proteins (HIPPs) in rice (*Oryza sativa*) contain HMA domains, and have been identified as putative effector hubs (Bentham *et al.* 2020; Maidment *et al.* 2021) as HIPPs have been shown to be the target of multiple fungal effector proteins, having a greater binding affinity to *M. oryzae* AVR-Pik variants than the integrated HMA domains present in rice CC-NLR resistance genes *Pik-1* and *Pik-2* (Maidment *et al.* 2021).

Importantly, HMA domain variants have been shown to perceive new effectors (Césari *et al.* 2022). On haplotype 1, the genes Hap1_g26587 and Hap1_g26589 were given the functional description “heavy metal-associated isoprenylated plant protein 47” and are located 19 and 43 kb upstream, respectively, of the closest RGA4 on the minus strand. Conversely on haplotype 2, four HMA genes with the same descriptor (Hap2_g27459, Hap2_g27477, Hap2_g27482, and Hap2_g27484) were identified. These genes ranged from 20–72 kb away from the nearest putative RGA3 gene. HIPP genes of haplotypes 1 and 2, respectively, share high identity with minimal amino acid substitutions among each other. Performing a BLASTp of these predicted proteins against the entire protein set of both haplotypes resulted in matches with other predicted HIPPs, with no homology to suggest that the nearby RGA cluster has a unique synonymous integrated HMA domain like that in rice.

**Fig. 4.**
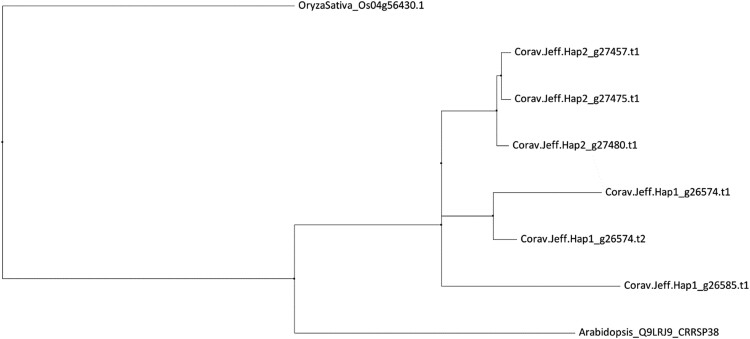
Neighbor joining tree of seven cysteine-rich secretory proteins (CRSPs) within the EFB R-gene region of both haplotypes with *Arabidopsis* and rice (*RCR3*) homologs aligned by MUSCLE.

In recent years it has become apparent that cysteine-rich receptor-like secreted proteins (CRRSPs) have crucial involvement in plant-fungal pathogen interactions (Zeiner i 2023). *Gnk2* from ginkgo (*Ginkgo biloba*) and two maize (*Zea mays*) proteins, *AFP1* and *AFP2*, bind to mannose during the defense response against fungal pathogens (Miyakawa *et al.* 2014; Ma *et al.* 2018). Mannose and its reduced sugar alcohol, mannitol, are independently important to both host plant and fungal pathogen metabolism and signaling during plant growth and pathogen invasion (Patel and Williamson 2016). CRRSPs have also been shown to be directly involved in fungal pathogen recognition as co-receptors for pathogen effectors (Wang *et al.* 2023). Recently *TaCRK3,* a CRRSP in wheat, was revealed to inhibit mycelial growth in vitro (Guo *et al.* 2021). Five genes were given the functional description “cysteine-rich repeat secretory protein 38”: two in haplotype 1, Hap1_g26574 and Hap1_g26585, and three in haplotype 2, Hap2_g27475, Hap2_g27480, and Hap2_g27457. To further investigate similarity between these CRRSPs, we performed a BLASTp and used MUSCLE to generate a neighbor-joining tree in JalView (Fig. 4).

**Fig. 5.**
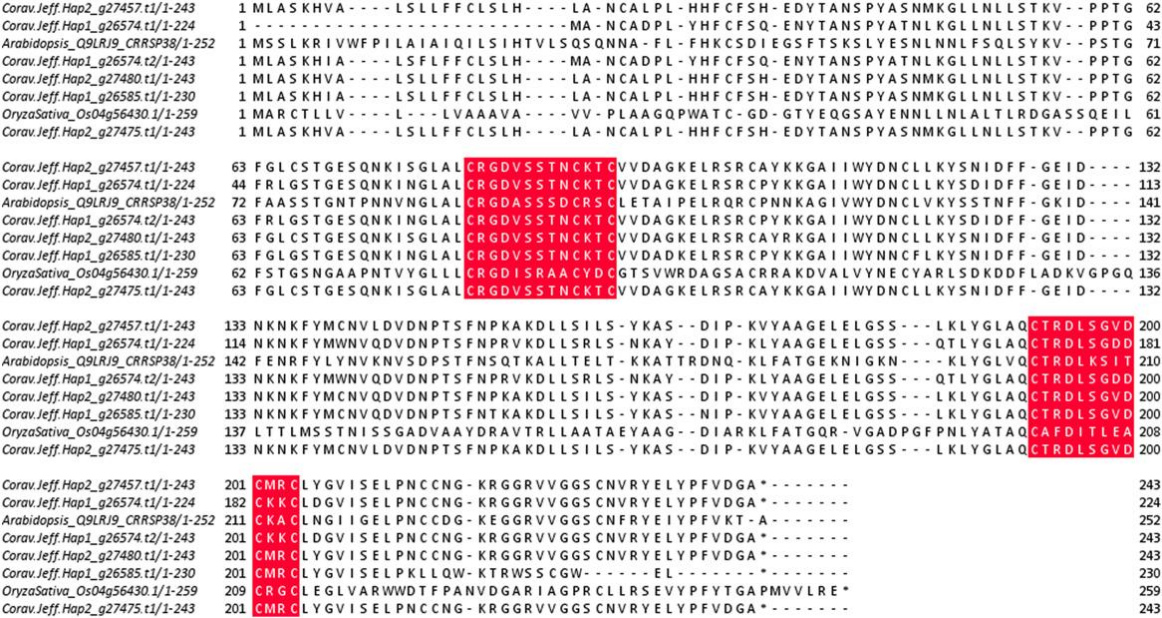
Amino-acid sequence alignment of all Cysteine-rich repeat secretory protein-38 in *C. avellana* “Jefferson” and a homolog from Arabidopsis and Rice (*RMC*). The numbers on the right side indicate the positions of the residues in the corresponding protein. Shading highlights the conserved motif of the DUF26 domain C-X8-C-X2-C.

The haplotype 1 gene Hap1_g26574 has two transcripts, with .t1 containing a 20 bp deletion at the 5' end; the two transcripts have an 86 and 88% similarity to the haplotype 1 gene (Hap1_g26585) and the haplotype 2 genes, respectively, whereas all haplotype 2 genes are 100% identical. These genes contained an extracellular domain composed of two DUF26 (domain of unknown function 26) motifs, but notably lacked an intracellular serine/threonine kinase domain and transmembrane domain (Fig. 5).

Despite identifying candidate EFB resistance genes on haplotype 1, the overall similarity between these genes and haplotype 2 R-genes makes it challenging to determine whether one or several resistance genes are involved in the activation of “Gasaway” resistance. It remains to be determined how the unique CRRSP (Hap1_g26574), with proximity to numerous NBS-LRRs, is involved in processes of pathogen detection and downstream signaling response. Thus, the uncharacterized disease resistance signaling pathway of “Gasaway” may involve NBS-LRR RGA homologs and CRRSPs, whereby pathotype specific effector(s) might target either a decoy or guardee. Further research is needed to characterize which haplotype 1 gene(s) are truly responsible for “Gasaway” EFB resistance and if the mechanism is based on a decoy (does not contribute to host susceptibility) or whether the alteration of the protein(s) results in enhanced susceptibility (guardee). The hazelnut breeding program at OSU has identified many sources of EFB resistance and sequenced select genomes to expand knowledge of the allelic diversity of putative resistance gene candidates. Examination of these EFB resistance sources may add additional evidence for the hypothesized molecular mechanism, with R-gene homologs acting in congruence with unique CRRSP proteins. Future work in determining EFB resistance mechanisms of other *C. avellana* cultivars should be based on comparisons between the pool of R-genes and CRRSP proteins derived from haplotype 1 of “Jefferson” to prospective EFB resistance genes to narrow the list of putative candidate genes.

## Conclusions

Here, we report the first haplotype-resolved chromosome-level genome assembly and annotation of the diploid *C. avellana* “Jefferson”. BUSCO analysis showed that the genome assemblies and structural annotations were of high quality. The ability of haplotype-phasing to identify parental genic contributions was successfully demonstrated by the complete separation of SI alleles to their respective parental haplotypes. Furthermore, the region associated with “Gasaway” EFB resistance was remapped with high confidence to the resistant parental haplotype, and several new candidate resistance genes were identified. The molecular mechanism behind “Gasaway” resistance remains to be investigated, however, the presence of the RGA cluster in congruence with a cysteine-rich secretory protein provides an attractive region to test disease model hypotheses. Detection of methylation around the SI and RGA clusters may further narrow the list of candidate genes, however, early users of the PacBio Sequel IIe platform did not have the option to obtain 5mC kinetics, so further “Jefferson” sequencing would be required to obtain this data. The haplotype-resolved “Jefferson” genome assembly and annotation presented here will serve as a powerful resource for hazelnut breeders and plant scientists in the further development of molecular markers for genomics-assisted breeding and facilitate future studies of *Corylus* biology and genetics.

## Supplementary Material

jkae021_Supplementary_Data

## Data Availability

The haplotype genome assemblies and annotations of *C. avellana* “Jefferson” presented here are available at the United States Department of Energy's Joint Genomics Institute Phytozome under genome ID 858 and 859. The “Jefferson” genome assembly, annotation, and respective read tracks, including the parental alignments, are also available as a genome browser via JBrowse2 at https://Hazelnutgenomes.oregonstate.edu/public. The raw sequence data is available on NCBI under BioProject PRJNA1107824. Scripts used for genome assembly can be found at: https://github.com/ViningLab/ViningLab_code/blob/main/G3_genomeAssemblyPipeline.txt. Supplemental material available at G3 online.
