# Supplementary material for: A haplotype-resolved chromosome-level assembly and annotation of European hazelnut (C. avellana cv. Jefferson) provides insight into mechanisms of eastern filbert blight resistance
Source: G3 (Bethesda). 2024 Feb 7;14(6):jkae021. doi: 10.1093/g3journal/jkae021 (PMC11152071; doi:10.1093/g3journal/jkae021)
Supplement: jkae021_Supplementary_Data [file jkae021_supplementary_data.zip › Supplemental_Tables_and_Figure_Legends_G3-2023-404767.docx]

**Supplemental**

**File S1. List of Supplementary Materials**

Table S1. Summary of sequencing data from Illumina, Hi-C, and PacBio platforms.

Table S2. Summary statistics for the eleven haplotype scaffolds corresponding to the ‘Jefferson’ European hazelnut (*C. avellana*) base chromosomes.

Table S3. Number and percentage of aligned Illumina 150 bp PE reads derived from ‘Jefferson’ parents to ‘Jefferson’ chromosome-level haplotype-resolved assemblies.

Table S4. ‘Jefferson’ haplotype 1 assembly EDTA output.

Table S5. ‘Jefferson’ haplotype 2 assembly EDTA output.

Table S6. Distribution of resistance-like genes identified by DRAGO2 among 11 pseudo-chromosomal scaffolds of the ‘Jefferson’ haplotype 1 and haplotype 2 assemblies.

Figure S1**.** Genome assembly and annotation workflow of *C. avellana* ‘Jefferson’.

Figure S2. GenomeScope of raw ‘Jefferson’ PacBio HiFi reads with k-mer length = 31.

Figure S3. LAI scores of *C. avellana* ‘Jefferson’ haplotypes and ‘Tombul’.

Figure S4. OmicsBox summary metrics of ‘Jefferson’ haplotype 1 functional annotation.

Figure S5. OmicsBox summary metrics of ‘Jefferson’ haplotype 2 functional annotation.

**Table S1** Summary of sequencing data from Illumina, Hi-C, and PacBio platforms.

| **Sequencing platform** | **Sample** | **Insert length** | **Sequencing model** | **Number of reads** | **Total nucleotides** |
| --- | --- | --- | --- | --- | --- |
| PacBio Sequel IIe | ‘Jefferson’ | >20kb | 2x 8M SMRT cell | 3.64 M | 56.8 Gb |
| Hiseq 4000 | ‘OSU 252.146’ | 300bp | 2x150 | 295.9 M | 44.38 Gb |
| Hiseq 4000 | ‘OSU 414.062’ | 300bp | 2x150 | 218.2 M | 32.73 Gb |
| Dovetail Hi-C on Hiseq 4000 | ‘Jefferson’ | 300bp | 2x150 | 428.46 M | 64.69 Gb |

**Table S2**. Summary statistics for the eleven haplotype scaffolds corresponding to the ‘Jefferson’ European hazelnut (*C. avellana*) base chromosomes.

| **Chromosomes** | **Haplotype 1** | | | | **Haplotype 2** | | | |
| --- | --- | --- | --- | --- | --- | --- | --- | --- |
|  | Total length (bp) | N count^1^ | | Gaps | | Total length  (bp) | N count^1^ | Gaps |
| 1 | 48,258,603 | | 600 | 3 | 47,666,154 | | 400 | 2 |
| 2 | 44,374,200 | | 200 | 1 | 45,407,320 | | 600 | 3 |
| 3 | 33,425,378 | | 200 | 1 | 32,429,289 | | 0 | 0 |
| 4 | 36,823,049 | | 1,200 | 6 | 37,439,655 | | 400 | 2 |
| 5 | 32,584,664 | | 400 | 2 | 35,167,529 | | 800 | 4 |
| 6 | 28,787,360 | | 0 | 0 | 28,771,021 | | 200 | 1 |
| 7 | 31,176,844 | | 200 | 1 | 31,107,465 | | 200 | 1 |
| 8 | 24,916,583 | | 400 | 2 | 23,719,546 | | 200 | 1 |
| 9 | 23,914,400 | | 400 | 2 | 23,029,850 | | 800 | 4 |
| 10 | 23,820,452 | | 200 | 1 | 24,758,360 | | 400 | 2 |
| 11 | 21,620,711 | | 600 | 3 | 22,513,321 | | 200 | 1 |
| **Total genome size** | **349,702,244** | | **4,400** | **22** | **352,009,510** | | **4,200** | **21** |

^1^Ns are inserted by YaHs at a fixed rate of 200 nucleotides for every contig merge.

**Table S3.** Number and percentage of aligned Illumina 150 bp PE reads derived from ‘Jefferson’ parents to ‘Jefferson’ chromosome-level haplotype-resolved assemblies.

|  | **Number and percentage of aligned reads for each parent** | |
| --- | --- | --- |
|  | ‘OSU 252.146’ | ‘OSU 414.062’ |
| ‘Jefferson’ V4 Haplotype 1 | 267,993,778 (90.57%) | 200,943,531 (92.08%) |
| ‘Jefferson’ V4 Haplotype 2 | 272,354,026 (92.04%) | 199,451,255 (91.39%) |

**Table S4.** ‘Jefferson’ haplotype 1 assembly EDTA^1^ output.

| Class | Number of elements | Length (bp) | Percentage of genome |
| --- | --- | --- | --- |
| **LTR** | **103,937** | **62,391,913** | **17.84%** |
| Copia | 23,301 | 15,899,955 | 4.55% |
| Gypsy  unknown | 25,625  55,011 | 21,135,697  25,356,261 | 6.04%  7.25% |
| **TIR** | **158,209** | **40,419,512** | **11.55%** |
| CACTA | 33,740 | 9,654,395 | 2.76% |
| Mutator | 77,114 | 17,487,304 | 5.00% |
| PIF_Harbinger | 22,873 | 5,671,550 | 1.62% |
| Tc1_Mariner | 3,700 | 878,845 | 0.25% |
| hAT | 20,782 | 6,727,418 | 1.92% |
| **nonLTR** | **1,065** | **361,136** | **0.10%** |
| LINE_element | 1,031 | 352,462 | 0.10% |
| unknown | 34 | 8,674 | 0.00% |
| **nonTIR** | -- | -- | -- |
| helitron | **35,911** | **9,497,804** | **2.72%** |
| **repeat_region** | **81,361** | **21,122,389** | **6.04%** |
|  | | | |
| **Total Genome Masked** | **380,483** | **133,792,754** | **38.26%** |

**^1^**EDTA run with parameters: --cds --bed --sensitive 1 --analysis 1; the CDS and bed file provide is derived from the BRAKER1/BRAKER2 gene set produced for the respective haplotype.

**Table S5.** ‘Jefferson’ haplotype 2 assembly EDTA^1^ output.

| Class | Number of elements | Length (bp) | Percentage of genome |
| --- | --- | --- | --- |
| **LTR** | **126,989** | **67,657,376** | **19.22%** |
| Copia | 27,892 | 17,200,312 | 4.89% |
| Gypsy  unknown | 26,884  72,213 | 21,861,582  28,595,482 | 6.21%  8.12% |
| **TIR** | **145,984** | **37,854,874** | **10.75%** |
| CACTA | 27,535 | 7,250,762 | 2.06% |
| Mutator | 74,679 | 18,295,776 | 5.20% |
| PIF_Harbinger | 17,714 | 4,238,613 | 1.20% |
| Tc1_Mariner | 3,122 | 681,523 | 0.19% |
| hAT | 22,934 | 7,388,200 | 2.10% |
| **nonLTR** | **1,100** | **442,005** | **0.12%** |
| LINE_element | 1,047 | 425,994 | 0.12% |
| unknown | 53 | 16,011 | 0.00% |
| **nonTIR** | -- | -- | -- |
| helitron | **39,521** | **8,684,384** | **2.47%** |
| **repeat_region** | **37,689** | **9,592,939** | **2.73%** |
|  | | | |
| **Total Genome Masked** | **380,483** | **124,231,578** | **35.29%** |

**^1^**EDTA run with parameters: --cds --bed --sensitive 1 --analysis 1; the CDS and bed file provide is derived from the BRAKER1/BRAKER2 gene set produced for the respective haplotype.

**Table S6.** Distribution of resistance-like transcripts identified by DRAGO2 among 11 pseudo-chromosomal scaffolds of the ‘Jefferson’ haplotype 1 and haplotype 2 assemblies.

| **‘Jefferson’ Pseudo-chromosomal scaffolds** | **CN^1^** | **CNL^1^** | **NL^1^** | **RLK^2^** | **RLP^2^** | **TN^3^** | **TNL^3^** | **Other^4^** | **Total** |
| --- | --- | --- | --- | --- | --- | --- | --- | --- | --- |
|  | H1/H2 | H1/H2 | H1/H2 | H1/H2 | H1/H2 | H1/H2 | H1/H2 | H1/H2 | H1/H2 |
| 1 | 17/16 | 63/60 | 43/47 | 20/17 | 29/32 | 0/2 | 8/5 | 304/276 | 484/455 |
| 2 | 18/23 | 12/14 | 22/28 | 84/65 | 91/58 | 3/1 | 0/0 | 383/403 | 613/592 |
| 3 | 4/5 | 5/5 | 8/7 | 36/36 | 28/25 | 3/5 | 14/11 | 162/169 | 260/263 |
| 4 | 3/10 | 14/8 | 1/4 | 50/57 | 24/24 | 0/0 | 1/1 | 189/207 | 284/315 |
| 5 | 3/1 | 4/0 | 12/11 | 41/40 | 43/23 | 5/2 | 6/6 | 224/225 | 342/298 |
| 6 | 1/2 | 2/1 | 4/5 | 45/49 | 15/31 | 15/13 | 21/15 | 233/240 | 335/356 |
| 7 | 0/0 | 5/5 | 2/1 | 66/71 | 44/62 | 0/0 | 0/0 | 165/179 | 284/320 |
| 8 | 5/8 | 11/20 | 5/5 | 39/37 | 70/58 | 14/10 | 47/31 | 166/169 | 360/345 |
| 9 | 11/14 | 10/9 | 3/6 | 15/21 | 16/21 | 1/1 | 12/10 | 126/128 | 196/211 |
| 10 | 2/1 | 11/9 | 6/2 | 45/50 | 63/66 | 0/0 | 2/3 | 136/147 | 265/280 |
| 11 | 0/0 | 2/2 | 0/0 | 45/45 | 22/23 | 0/0 | 0/0 | 127/154 | 197/224 |
| **Total** | **66/80** | **139/133** | **117/122** | **486/445** | **445/ 423** | **41/34** | **111/82** | **2,215/**  **2,297** | **3,620/**  **3,659** |

^1^Coiled-coil nucleotide binding site [(CC-NBS (CN)]; CC-NBS-leucine rich repeat [(CC-NBS-LRR (CNL)]; NBS-LRR (NL).

^2^Receptor-like Kinase (RLK); Receptor-like Protein (RLP).

^3^TIR-NBS (TN); TIR-NBS-LRR (TNL).

^4^Includes kinases (K), NBS (N), LRRs (L), CKs, CTs, CTLs, Lysine motif containing proteins (LYK and LYP) and Lectin-like motif containing proteins (LECM).

**Figure S1.** Genome assembly and annotation workflow of *C. avellana* ‘Jefferson.’ Figure shows the genome assembly and annotation pipeline with processes shown in green, extraneous data in orange and quality checks in blue.

**Figure S2.** GenomeScope result of raw ‘Jefferson’ PacBio HiFi reads for k-mer length = 21. GenomeScope output derived from jellyfish count -C -m 21 -s 1000000000 and jellyfish histo.

**Figure S3.** LAI scores of *C. avellana* ‘Jefferson’ haplotypes and ‘Tombul.’ LAI scores were obtained by LTR_Retriever from a concatenated set of LTRs derived from LTR harvest and LTR_FINDER_parallel for each respective assembly. Each dot represents LAI score of a 3 Mb-sliding window with 300-Kb increment, adjusted by the whole-genome LTR identity. (**A**) ‘Jefferson’ haplotype 1 genome assembly. (**B**) ‘Jefferson’ haplotype 2 genome assembly. (**C**) ‘Tombul’ genome assembly.

**Figure S4.** OmicsBox summary metrics of ‘Jefferson’ haplotype 1 functional annotation. Pie chart shows total distribution of OmicsBox functional annotation performed on haplotype 1 amino acid transcripts of ‘Jefferson’. In red are transcripts that received no BLAST hits from the database and thus have unknown function; orange are transcripts that received only BLAST hits; green are transcripts that had GO terms associated with the initial BLAST database search; blue is transcripts that received GO annotation descriptions.

**Figure S5.** OmicsBox summary metrics of ‘Jefferson’ haplotype 2 functional annotation. Pie chart shows total distribution of OmicsBox functional annotation performed on haplotype 2 amino acid transcripts of ‘Jefferson’.
