# Supplementary figures and images for: A haplotype-resolved chromosome-level assembly and annotation of European hazelnut (C. avellana cv. Jefferson) provides insight into mechanisms of eastern filbert blight resistance
Source: G3 (Bethesda). 2024 Feb 7;14(6):jkae021. doi: 10.1093/g3journal/jkae021 (PMC11152071; doi:10.1093/g3journal/jkae021)

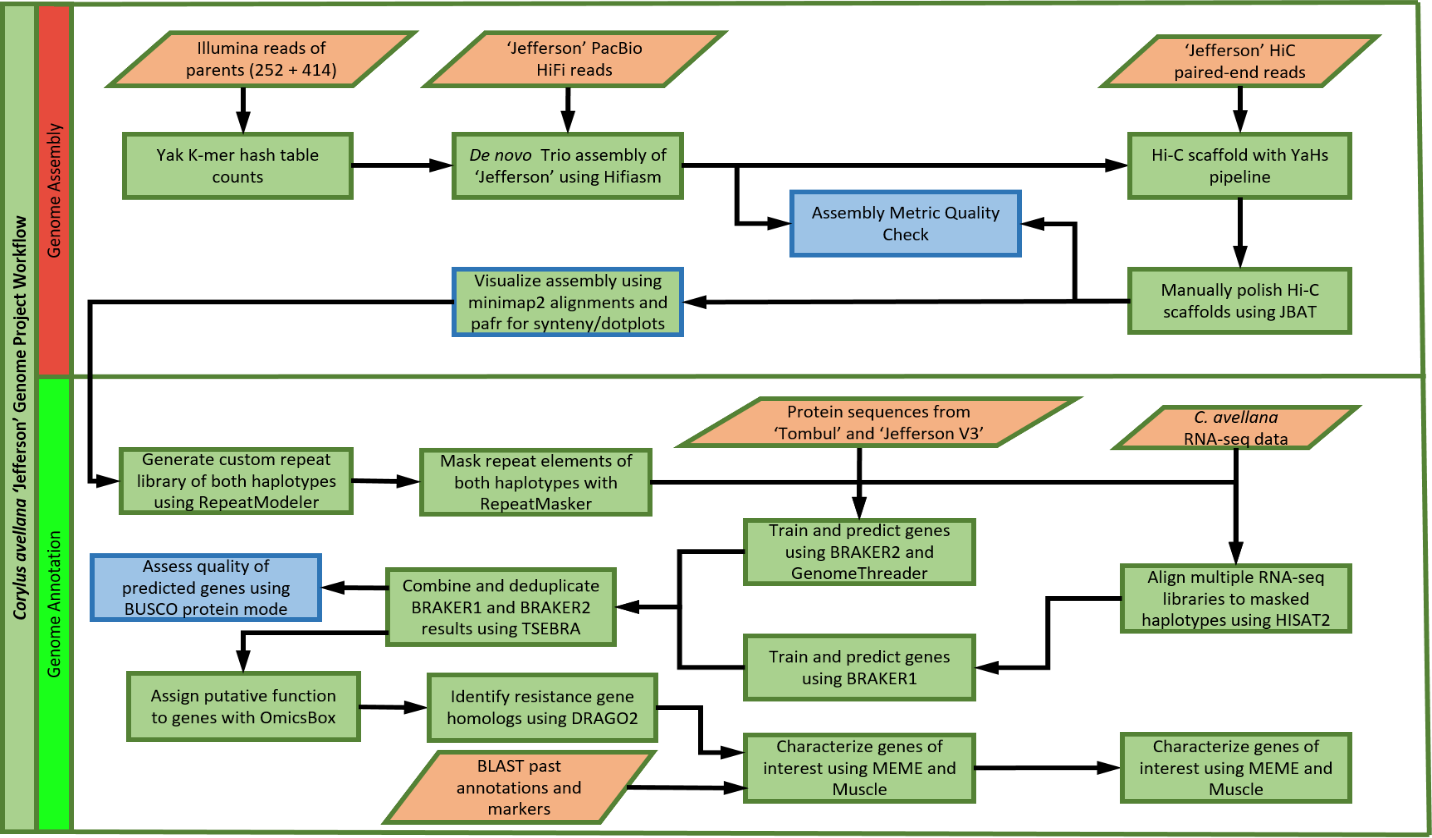

Supplement: jkae021_Supplementary_Data [file jkae021_supplementary_data.zip › Figure_S1_G3-2023-404767.tif]

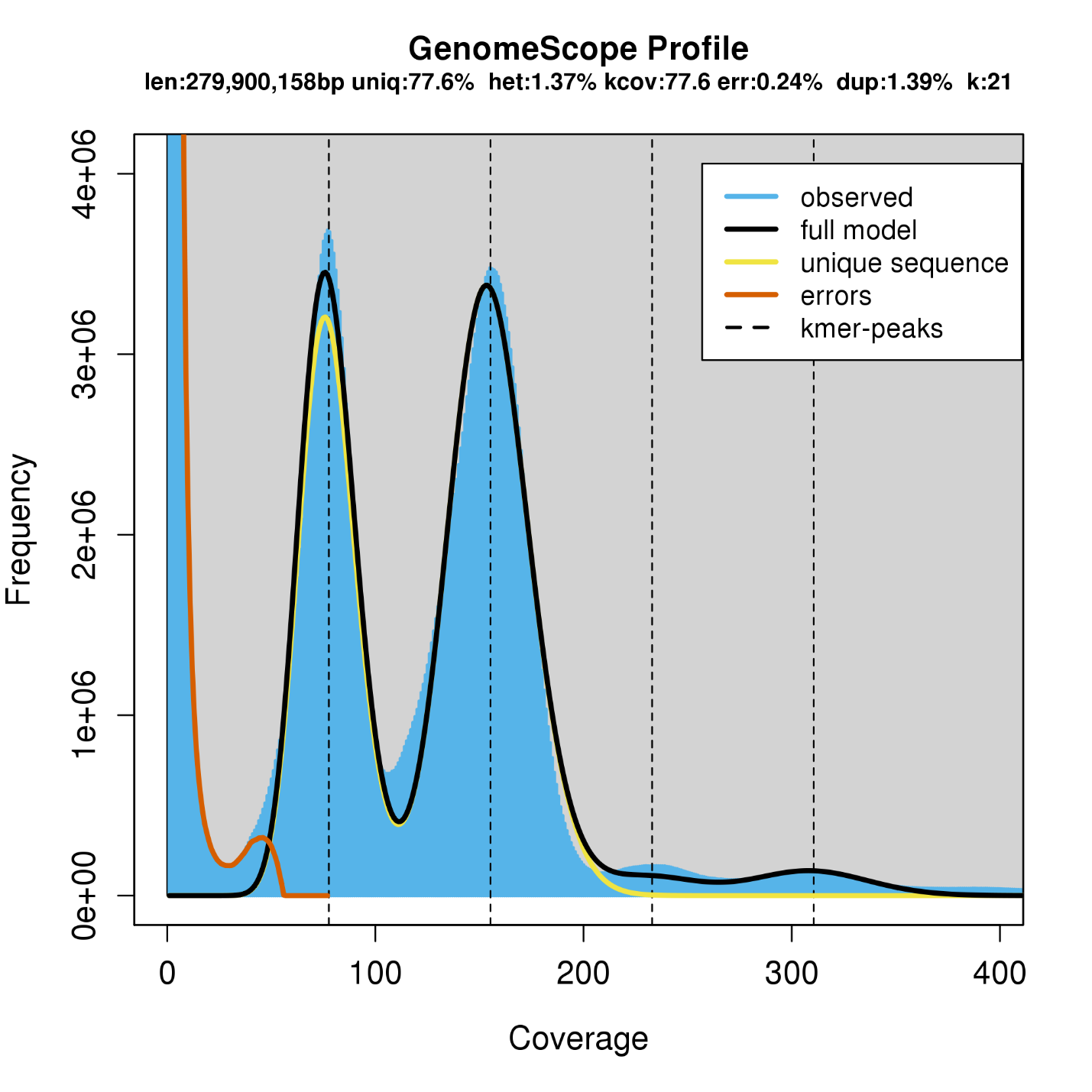

Supplement: jkae021_Supplementary_Data [file jkae021_supplementary_data.zip › Figure_S2_G3-2023-404767.tif]

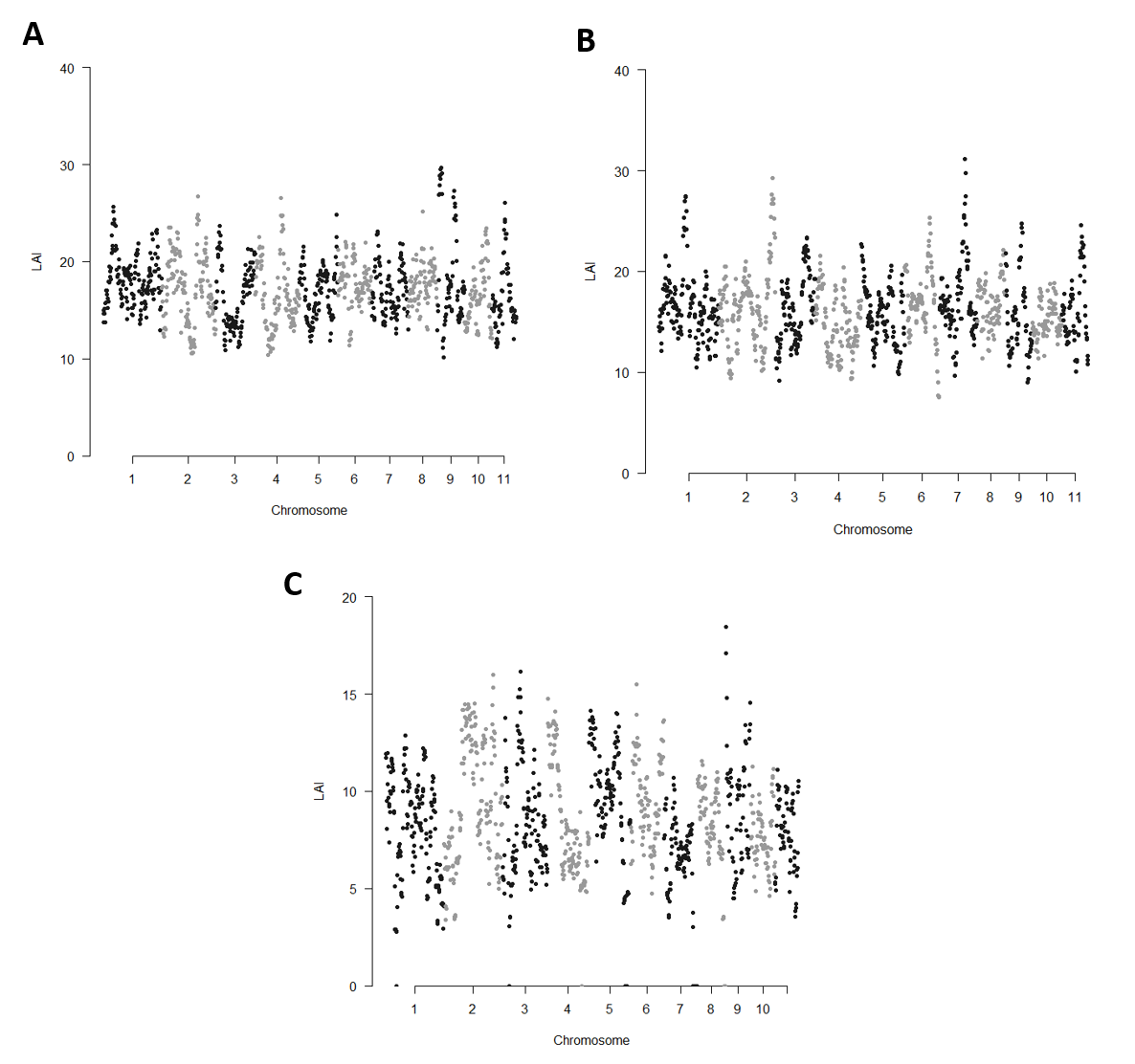

Supplement: jkae021_Supplementary_Data [file jkae021_supplementary_data.zip › Figure_S3_G3-2023-404767.tif]

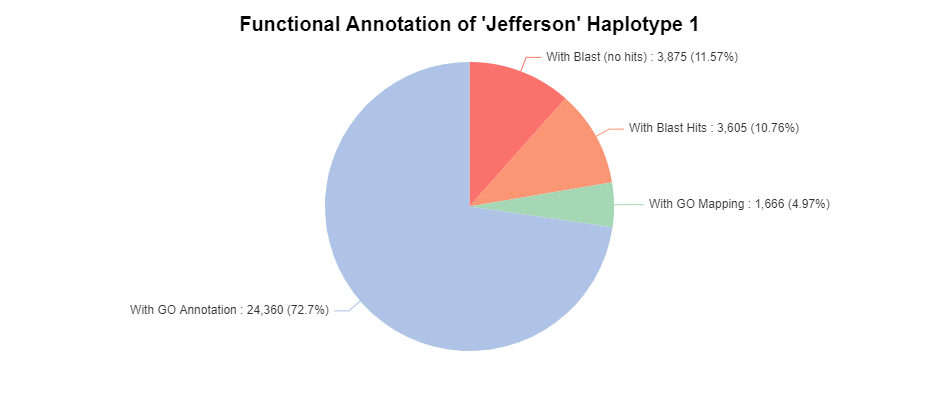

Supplement: jkae021_Supplementary_Data [file jkae021_supplementary_data.zip › Figure_S4_G3-2023-404767.tif]

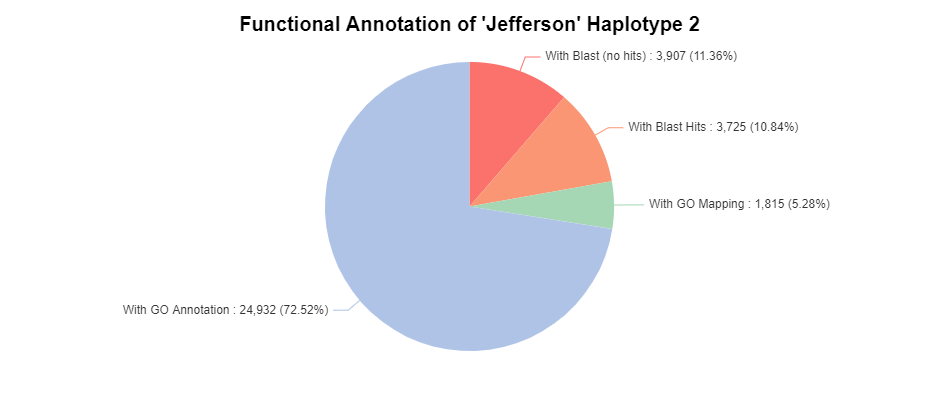

Supplement: jkae021_Supplementary_Data [file jkae021_supplementary_data.zip › Figure_S5_G3-2023-404767.tif]
